# Supplementary material for: An annotated list of bivalent chromatin regions in human ES cells: a new tool for cancer epigenetic research
Source: Oncotarget. 2016 Dec 1;8(3):4110–24. doi: 10.18632/oncotarget.13746 (PMC5354816; doi:10.18632/oncotarget.13746)
Supplement: Supplementary file 1 [file oncotarget-08-4110-s001.pdf]

An annotated list of bivalent chromatin regions in human ES cells: a new tool for cancer epigenetic research

Supplementary Materials

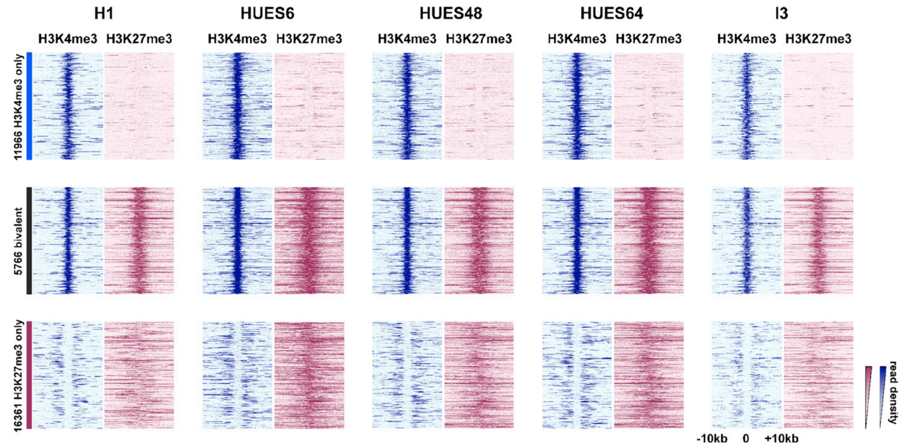

Supplementary Figure S1: H3K27me3 and H3K4me3 enrichment at bivalent, H3K4me3- and H3K27me3-only regions. Chip-seq read density for H3K27me3 (purple) and H3K4me3 (blue) at high-confidence H3K4me3-only, bivalent and H3K27me3-only regions in each hESC line. Each line represents one genomic region. A −10Kb to +10 Kb window centered on the peak of the signal is shown.

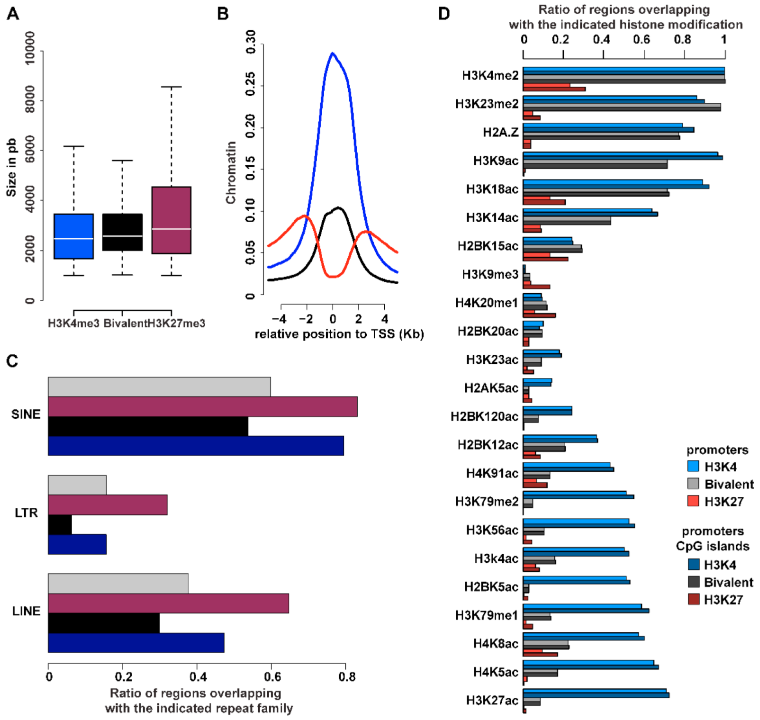

Supplementary Figure S2: Features of high-confidence H3K4me3-only, bivalent and KH3K27me3-only regions. (**A**) Distribution of the region size for H3K4me3-only (blue); bivalent (black) and H3K27me3-only (purple) high-confidence genomic regions. (**B**) Relative distance from the TSS of H3K4me3-only (blue), bivalent (black) and H3K27-only (red) regions associated with promoters. (**C**) Proportion of all (grey line), H3K4me3-only (blue line), bivalent (black line) and H3K27me3-only (purple line) promoters (−1Kbp and +1Kbp from TSS) containing SINE (upper panel), LTR (middle panel) and LINE (lower panel) retroelements. (**D**) Proportion of promoters and CpG island-rich promoters, enriched for the indicated histone marks, according to their H3K4me3- (light and dark blue), bivalent (light and dark grey) or H3K27me3-only (light and dark red) signature.

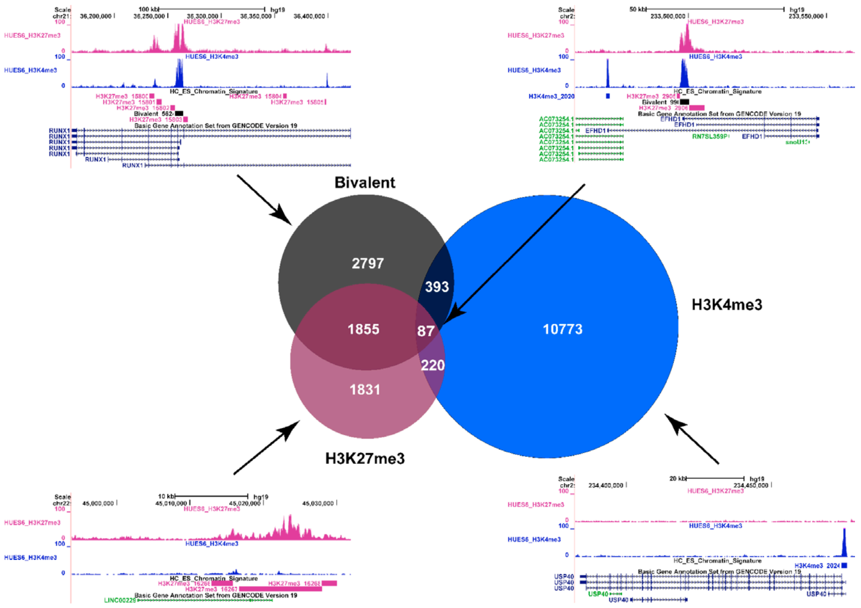

Supplementary Figure S3: Chromatin signatures at genes with several promoters. To determine the gene ontology term enrichment, genes with several promoters were filtered to retain only those with a similar chromatin signature at all their marked promoters. *RUNX1*, *LINC00229* and *USP40* are shown as examples of genes associated with bivalent, H3K4me3- and H3K27me3-only promoters, respectively. By contrast, *EFDH1* displays several signatures according to the splice-variant promoter studied (from 5′ to 3′: H3K4me3-only, bivalent and H3K27me3-only). This gene type was not retained for the GO and transcriptional analyses. Note that when a gene contains also a splice-variant promoter without any of these three signatures (i.e., *RUNX1* or *USP40*), only the “marked” promoters were considered.

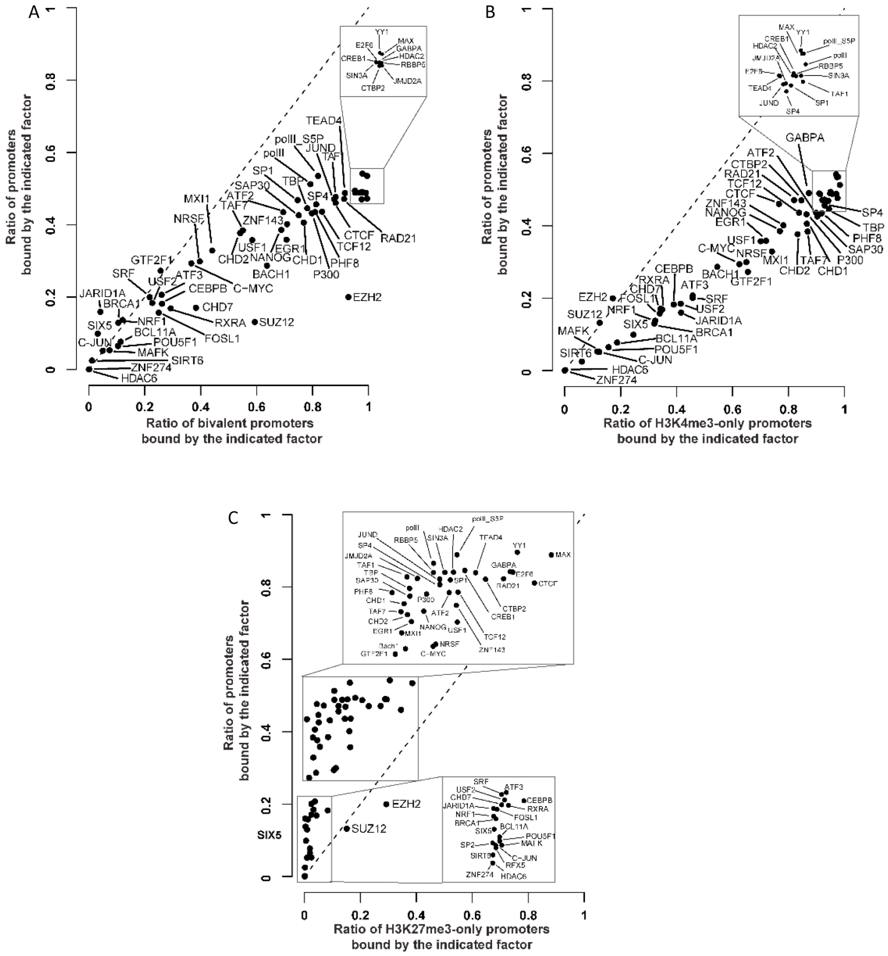

Supplementary Figure S4: Transcription factor occupancy at promoters. Transcription factors occupancy at bivalent (**A**), H3K4me3-only (**B**) and H3K27me3-only (**C**) promoters versus all promoters.

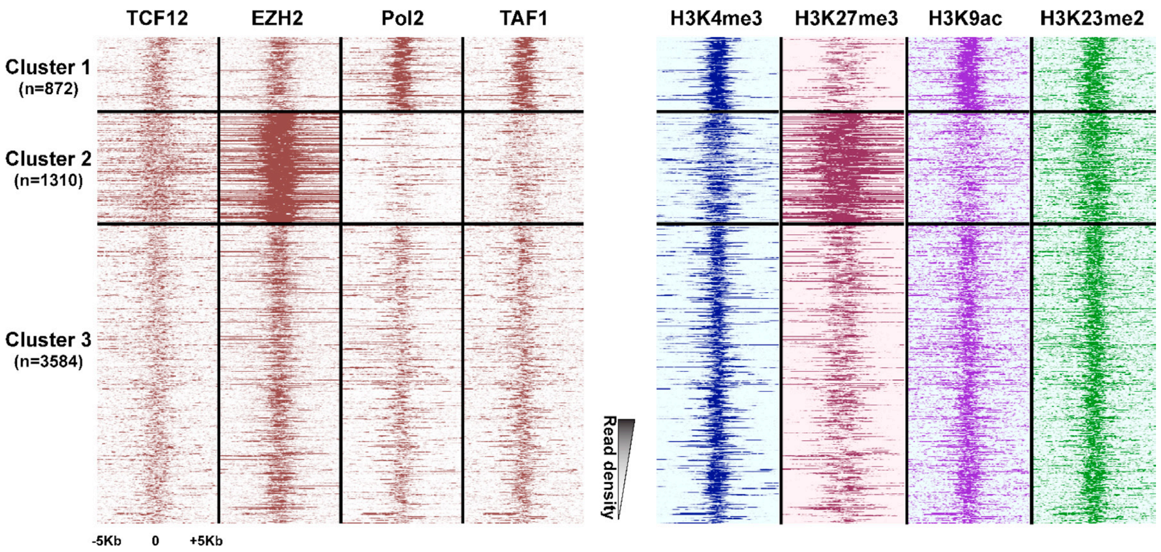

Supplementary Figure S5: Three clusters of high-confidence bivalent regions in hESCs. All bivalent regions were classified in three clusters based on the extent and density of the EZH2, PolII, TAF1 and TCF-12 signals. Cluster 2 is characterized by a strong and extended signal for EZH2 and H3K27me3.

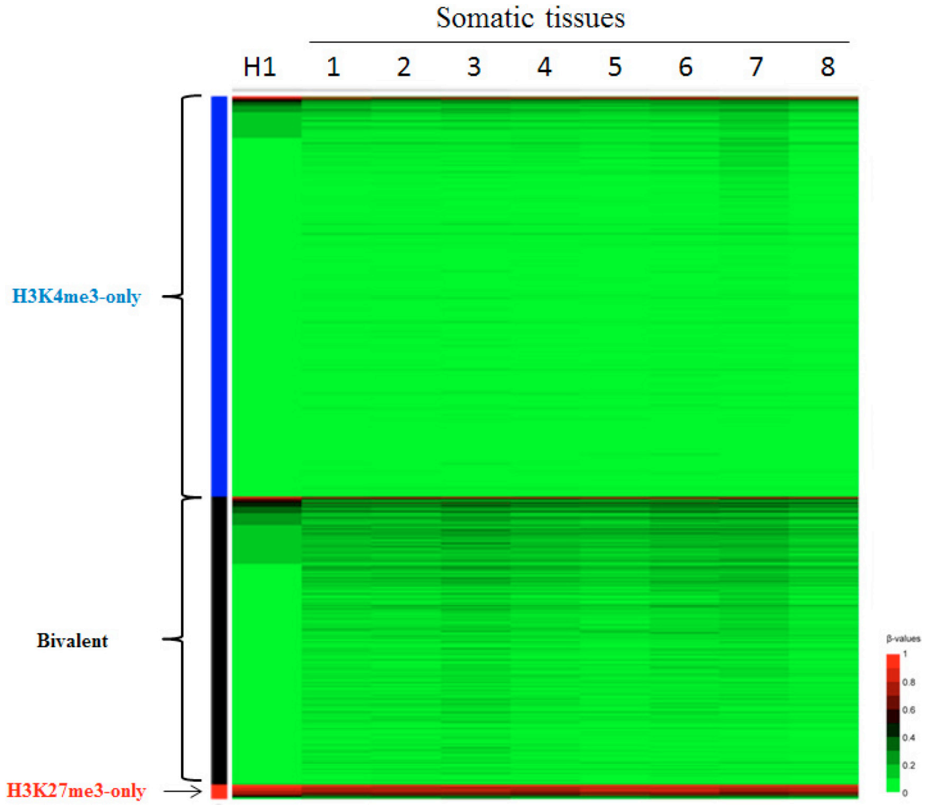

Supplementary Figure S6: CGIs DNA methylation pattern observed in hESC is globally maintained in somatic tissues. Methylation level of CGIs (β-values) in H1 hESCs and the somatic tissues used as tumor samples matched control, according to their chromatin signature in hESC. The overall DNA methylation pattern observed in hESCs is maintained in normal tissues with the majority of CGIs marked by bivalency and H3K4me3-only in hESCs remaining unmethylated. Somatic tissues analyzed are the normal matched control of (1) BLCA: bladder urothelial carcinoma; (2) BRCA: breast invasive carcinoma; (3) COAD: colon adenocarcinoma; (4) HNSC: head-neck squamous cell carcinoma; KIRP: (5) kidney renal papillary cell carcinoma; (6) LIHC: liver hepatocellular carcinoma; (7) LUSC: lung squamous cell carcinoma and (8) LUAD: lung adenocarcinoma tumor type, respectively

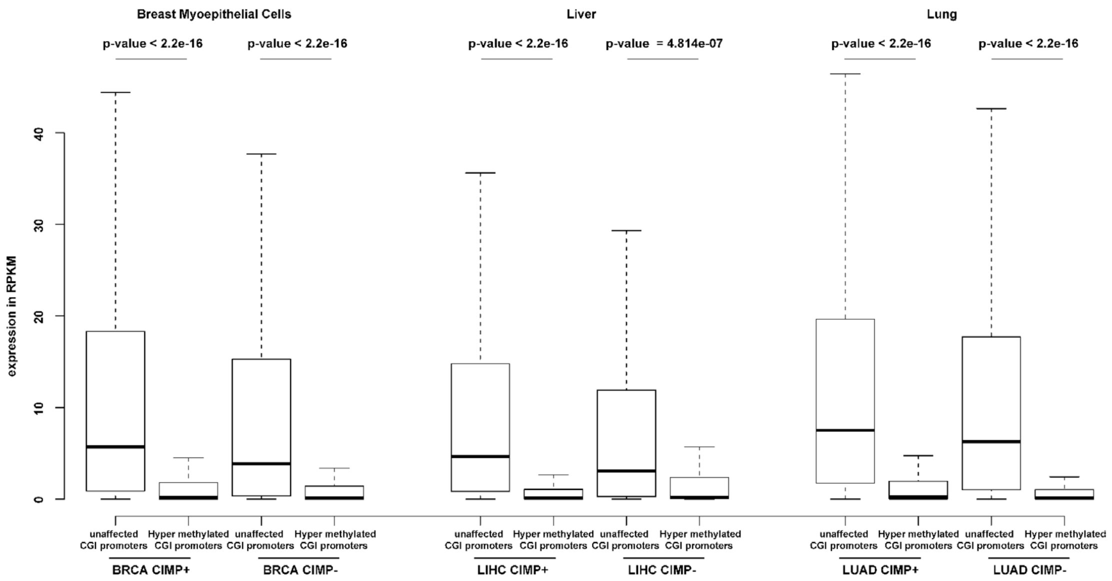

Supplementary Figure S7: Genes with aberrantly hypermethylated CGI/promoter in tumors tend to be repressed in the corresponding healthy tissue. Boxplots of the expression levels measured in healthy tissues of genes with unaffected or hypermethylated CGI/promoter in the corresponding CIMP-positive (+) and CIMP-negative (−) tumors (*p*-value: Mann-Whitney test). BRCA: breast invasive carcinoma; LIHC: liver hepatocellular carcinoma; LUAD: lung adenocarcinoma.

Supplementary Table S1: Details of the datasets used in this study (obtained from the NIH Roadmap Epigenomics project http://www.ncbi.nlm.nih.gov/geo/roadmap/epigenomics/)

Cell line	Series	Experiment	Dataset	Medium	Antibody
ES H1	Dataset series 1	input	GSM433179	TESR	
		H3K27me3	GSM537683	TESR	Millipore 07–449
		H3K4me3	GSM537681	TESR	Millipore 07–473
	Dataset series 2	input	GSM605335	mTeSER	
		H3K27me3	GSM605308	mTeSER	Millipore 07–449
		H3K4me3	GSM605315	mTeSER	Millipore 04–745
	Dataset series 3	input	GSM605339	mTeSER	
		H3K27me3	GSM466734	mTeSER	Upstate 07–449
		H3K4me3	GSM469971	mTeSER	Abcam ab8580
ES –I3	Dataset series 4	input	GSM537647	KSR	
		H3K27me3	GSM537648	KSR	Millipore 07–449
		H3K4me3	GSM537665	KSR	Millipore 07–473
	Dataset series 5	input	GSM621386	TESR	
		H3K27me3	GSM537627	TESR	Millipore 07–449
		H3K4me3	GSM537626	TESR	Millipore 07–473
HUES64	Dataset series 6	input	GSM772754	KSR	
		H3K27me3	GSM772750	KSR	Millipore 07–449
		H3K4me3	GSM772752	KSR	Millipore 07–473
	Dataset series 7	input	GSM772807	KSR	
		H3K27me3	GSM669974	KSR	Millipore 07–449
		H3K4me3	GSM669967	KSR	Millipore 07–473
HUES6	Dataset series 8	input	GSM669888	KSR	
		H3K27me3	GSM669887	KSR	Millipore 07–449
		H3K4me3	GSM669889	KSR	Millipore 07–473
	Dataset series 9	input	GSM669895	KSR	
		H3K27me3	GSM669897	KSR	Millipore 07–449
		H3K4me3	GSM669893	KSR	Millipore 07–473
HUES48	Dataset series 10	input	GSM772755	KSR	
		H3K27me3	GSM669942	KSR	Millipore 07–449
		H3K4me3	GSM669936	KSR	Millipore 07–473
	Dataset series 11	input	GSM772794	KSR	
		H3K27me3	GSM772766	KSR	Millipore 07–449
		H3K4me3	GSM772797	KSR	Millipore 07–473

Supplementary Table S2: Number of ChIP-seq tags after filtering, and peak calling parameters for each hESC line used in this study. See Supplemenatry_Table_S2

Supplementary Table S3: Results of peak calling analyses for each hESC line

hESC line	Histone mark	Total peaks	Peaks wider than 1kb	Total bivalent	Bivalent identified	Bivalent cell-specific
**ES H1**	H3K27me3	80064	21447	12402	7783	239
	H3K4me3	26354	19390			
**ES -I3**	H3K27me3	92099	20713		7756	283
	H3K4me3	50312	25650			
**HUES64**	H3K27me3	60580	25288		9824	585
	H3K4me3	34488	26204			
**HUES6**	H3K27me3	73140	41727		10266	1031
	H3K4me3	39677	29132			
**HUES48**	H3K27me3	56163	23971		8508	239
	H3K4me3	43417	28581			

Supplementary Table S4: Genomic features associated with H3K4me3-only, bivalent and H3K27me3-only regions

		**H3K4me3-only**		**Bivalent**		**H3K27me3-only**	
		Number	% of total	Number	% of total	Number	% of total
**CpG islands**	Promoter	7295	61.0%	4080	70.8%	248	1.5%
	Gene body	245	2.0%	491	8.5%	228	1.4%
	Intergenic	262	2.2%	658	11.4%	164	1.0%
	**Total CpG islands**	7802	65.2%	5229	90.7%	640	3.9%
**Non-CpG islands**	Promoter	1578	13.2%	250	4.3%	3775	23.1%
	Gene body	1417	11.8%	96	1.7%	5653	34.6%
	Intergenic	1169	9.8%	191	3.3%	6293	38.5%
	**Total non-CpG islands**	4164	34.8%	537	9.3%	15721	96.1%
	**Total**	**11966**	**100%**	**5766**	**100%**	**16361**	**100%**

Supplementary Table S5: ChIP-seq datasets for the histone modifications used in this study. (98 + 10 datasets from GSE17312, GSE16368 or GSE16256). See Supplemenatry_Table_S5.

Supplementary Table S6: ChIP-seq datasets for transcription factors in the hESC line H1 used in this study. See Supplemenatry_Table_S6.

Supplementary Table S7: Details of the coverage for the EPIC (upper table) and HM450K (lower table) arrays. HC, high confidence. See Supplemenatry_Table_S7.

Supplementary Table S8: Datasets extracted from the TCGA data portal for cancer methylation analyses. BLCA = bladder urothelial carcinoma; BRCA = breast invasive carcinoma; COAD = colon adenocarcinoma; HNSC = head-neck squamous cell carcinoma; KIRP = kidney renal papillary cell carcinoma; LIHC = liver hepatocellular carcinoma; LUSC = lung squamous cell carcinoma; LUAD = lung adenocarcinoma; NT = Normal Matched Tumor; TN = Tumor Matched Normal. See Supplemenatry_Table_S8.

Supplementary Table S9: Details on CIMP status definition for each tumor type

	number of HM450K probes defining the CIMP status	number of associated loci (CGI)	Proportion of CGI in bivalent-only regions	Proportion of CGI in H3K4me3-only regions	Proportion of CGI in H3K27me3-only regions
Bladder urothelial carcinoma (BLCA)	338	242	66,5%	8,7%	0,8%
Breast invasive carcinoma (BRCA)	1311	852	74,1%	4,5%	1,1%
Colon adenocarcinoma (COAD)	2656	1252	74,0%	9,2%	0,2%
Head-neck squamous cell carcinoma (HNSC)	1228	728	70,1%	10,6%	0,4%
Kidney renal papillary cell carcinoma (KIRP)	40	36	52,8%	8,3%	8,3%
Liver hepatocellular carcinoma (LIHC)	544	382	62,3%	13,1%	0,0%
Lung adenocarcinoma (LUAD)	1667	998	73,0%	2,7%	1,5%
Lung squamous cell carcinoma (LUSC)	1430	855	66,0%	5,7%	1,5%

HM450K probes defining the CIMP status are issued from [45]. The vast majority of CGI defining the CIMP status are marked by a bivalent signature in hES cells.

Supplementary Table S10: Distribution (percentage) of hyper- (upper table) and hypo- (lower table) methylated probes in CIMP-negative (−) and CIMP-positive (+) tumors (eight cancer types), according to the chromatin signature in hESCs. The number of affected genomic regions is indicated into brackets. BLCA: bladder urothelial carcinoma; BRCA: breast invasive carcinoma; COAD: colon adenocarcinoma; HNSC: head-neck squamous cell carcinoma; KIRP: kidney renal papillary cell carcinoma; LIHC: liver hepatocellular carcinoma; LUSC: lung squamous cell carcinoma; LUAD: lung adenocarcinoma. See Supplemenatry_Table_S10

Supplementary File S1: Annotated lists of high-confidence bivalent, H3K4me3-only and H3K27me3-only regions in hESC. See Supplementary_File_S1.

Supplementary File S2: Lists of high-confidence bivalent, H3K4me3-only and H3K27me3-only regions in hESC ready for uploading onto genome-browser. See Supplementary_File_S2.

Supplementary File S3: Gene ontology term enrichment of bivalent, H3K4me3-only and H3K27me3-only promoter regions See Supplementary_File_S3.

Supplementary File S4: Lists of high-confidence bivalent, H3K4me3-only and H3K27me3-only regions in hESC ready for uploading onto genome studio. See Supplementary_File_S4.
