# Supplementary material for: An annotated list of bivalent chromatin regions in human ES cells: a new tool for cancer epigenetic research
Source: Oncotarget. 2016 Dec 1;8(3):4110–24. doi: 10.18632/oncotarget.13746 (PMC5354816; doi:10.18632/oncotarget.13746)
Supplement: Supplementary file 2 [file oncotarget-08-4110-s002.docx]

| ES H1 | | | |
| --- | --- | --- | --- |
| Experiments | Datasets | Number of tags after filtering | Macs parameters |
| Input | GSM433179, GSM605335, GSM605339 | 42570628 | control |
| H3K27me3 | GSM466734, GSM605308, GSM537683 | 30295375 | shiftsize 73 p value 1e-3 |
| H3K4me3 | GSM469971, GSM537681, GSM605315 | 30677246 | shiftsize 73 p value 1e-5 |
| ES -I3 | | | |
| Input | GSM537647, GSM621386 | 43068256 | control |
| H3K27me3 | GSM537627, GSM537648 | 19479939 | shiftsize 73 p value 1e-3 |
| H3K4me3 | GSM537665, GSM537626 | 21553777 | shiftsize 73 p value 1e-5 |
| HUES64 | | | |
| Input | GSM772754, GSM772807 | 69183375 | control |
| H3K27me3 | GSM772750, GSM669974 | 43077527 | shiftsize 73 p value 1e-3 |
| H3K4me3 | GSM772752, GSM669967 | 51782114 | shiftsize 73 p value 1e-5 |
| HUES6 | | | |
| Input | GSM669888, GSM669895 | 67592902 | control |
| H3K27me3 | GSM669887, GSM669897 | 53364921 | shiftsize 73 p value 1e-3 |
| H3K4me3 | GSM669889, GSM669893 | 49413752 | shiftsize 73 p value 1e-5 |
| HUES48 | | | |
| Input | GSM772755, GSM772794 | 63864616 | control |
| H3K27me3 | GSM669942, GSM772766 | 44075742 | shiftsize 73 p value 1e-3 |
| H3K4me3 | GSM669936, GSM772797 | 46529022 | shiftsize 73 p value 1e-5 |

**Table S2: Number of ChIP-seq tags after filtering, and peak calling parameters for each hESC line used in this study.**
