# Supplementary material for: An annotated list of bivalent chromatin regions in human ES cells: a new tool for cancer epigenetic research
Source: Oncotarget. 2016 Dec 1;8(3):4110–24. doi: 10.18632/oncotarget.13746 (PMC5354816; doi:10.18632/oncotarget.13746)
Supplement: Supplementary file 3 [file oncotarget-08-4110-s003.docx]

| H1 | | | |
| --- | --- | --- | --- |
| Experiment | Datasets | Number of tags after filtering | Macs parameters |
| Input | GSM433179, GSM605335, GSM605339 | 42570628 | control |
| H3K27me3 | GSM466734, GSM605308, GSM537683 | 30295375 | shiftsize 73 p value 1e-3 -w |
| H3K4me3 | GSM469971, GSM537681, GSM605315 | 30677246 | shiftsize 73 p value 1e-5 -w |
| H3K4me2 | GSM602260, GSM602261 | 25165838 | shiftsize 73 p value 1e-5 -w |
| H4K91ac | GSM605332, GSM752991 | 45089153 | shiftsize 73 p value 1e-5 -w |
| H4K8ac | GSM896166, GSM908966 | 11212673 | shiftsize 73 p value 1e-5 -w |
| H4K5ac | GSM605330, GSM752990 | 39435745 | shiftsize 73 p value 1e-5 -w |
| H4K20me1 | GSM605329, GSM789284 | 27732642 | shiftsize 73 p value 1e-5 -w |
| H3K9me3 | GSM605327, GSM605328, GSM605325, GSM818057 | 58407470 | shiftsize 73 p value 1e-5 -w |
| H3K9ac | GSM605323, GSM410807, GSM433171, GSM434785, GSM537685 | 55685230 | shiftsize 73 p value 1e-5 -w |
| H3K79me2 | GSM605321, GSM605322 | 44983966 | shiftsize 73 p value 1e-5 -w |
| H3K79me1 | GSM605318, GSM605319, GSM605320 | 39142853 | shiftsize 73 p value 1e-5 -w |
| H3K56ac | GSM605317, GSM667627 | 33947972 | shiftsize 73 p value 1e-5 -w |
| H3K4ac | GSM605311, GSM667624 | 55930165 | shiftsize 73 p value 1e-5 -w |
| H3K27ac | GSM466732, GSM663427 | 21563659 | shiftsize 73 p value 1e-5 -w |
| H3K23me2 | GSM605305, GSM605306 | 37146838 | shiftsize 73 p value 1e-5 -w |
| H3K23ac | GSM667617, GSM667618 | 48501234 | shiftsize 73 p value 1e-5 -w |
| H3K18ac | GSM602259, GSM605304 | 27755027 | shiftsize 73 p value 1e-5 -w |
| H3K14ac | GSM667614, GSM667615 | 50429814 | shiftsize 73 p value 1e-5 -w |
| H2BK5ac | GSM605302, GSM605303 | 39829575 | shiftsize 73 p value 1e-5 -w |
| H2BK20ac | GSM605300, GSM605301 | 51320297 | shiftsize 73 p value 1e-5 -w |
| H2BK15ac | GSM605298, GSM605299 | 53381849 | shiftsize 73 p value 1e-5 -w |
| H2BK12ac | GSM605296, GSM605297 | 40711418 | shiftsize 73 p value 1e-5 -w |
| H2BK120ac | GSM605295, GSM789280, GSM789281 | 38929234 | shiftsize 73 p value 1e-5 -w |
| H2AK5ac | GSM602257, GSM602258 | 41018648 | shiftsize 73 p value 1e-5 -w |
| H2A.Z | GSM908938 | 8680395 | shiftsize 73 p value 1e-5 -w |

| H9 | | | |
| --- | --- | --- | --- |
| Experiment | Datasets | Number of tags after filtering | Macs parameters |
| Input | GSM667643, GSM706081, GSM832839 | 63828199 | control |
| H3K27me3 | GSM667622, GSM706066 | 43704839 | shiftsize 73 p value 1e-3 -w |
| H3K4me3 | GSM605316, GSM616128 | 23480130 | shiftsize 73 p value 1e-5 -w |
| H3K4me2 | GSM605314, GSM616127 | 38096043 | shiftsize 73 p value 1e-5 -w |
| H4K91ac | GSM667639, GSM667640 | 34280180 | shiftsize 73 p value 1e-5 -w |
| H4K8ac | GSM667637, GSM667638 | 39481348 | shiftsize 73 p value 1e-5 -w |
| H4K5ac | GSM667635, GSM667636 | 47193454 | shiftsize 73 p value 1e-5 -w |
| H4K20me1 | GSM667634 | 6349544 | shiftsize 73 p value 1e-5 -w |
| H3K9me3 | GSM667631, GSM667632, GSM667633 | 48687532 | shiftsize 73 p value 1e-5 -w |
| H3K9ac | GSM605324, GSM616129 | 36726256 | shiftsize 73 p value 1e-5 -w |
| H3K79me2 | GSM667630, GSM706078 | 51649507 | shiftsize 73 p value 1e-5 -w |
| H3K79me1 | GSM667629, GSM706077 | 33287074 | shiftsize 73 p value 1e-5 -w |
| H3K56ac | GSM667628, GSM706076 | 35130412 | shiftsize 73 p value 1e-5 -w |
| H3K4ac | GSM667625, GSM752976 | 46945617 | shiftsize 73 p value 1e-5 -w |
| H3K27ac | GSM605307, GSM665037 | 30721107 | shiftsize 73 p value 1e-5 -w |
| H3K23me2 | GSM667621, GSM807396 | 39611759 | shiftsize 73 p value 1e-5 -w |
| H3K23ac | GSM667619, GSM667620 | 32648665 | shiftsize 73 p value 1e-5 -w |
| H3K18ac | GSM667616, GSM706064 | 41904341 | shiftsize 73 p value 1e-5 -w |
| H3K14ac | GSM752964, GSM896161 | 36405933 | shiftsize 73 p value 1e-5 -w |
| H2BK5ac | GSM667612, GSM667613 | 59383140 | shiftsize 73 p value 1e-5 -w |
| H2BK20ac | GSM752963 | 11816696 | shiftsize 73 p value 1e-5 -w |
| H2BK15ac | GSM706062, GSM706063, GSM864034 | 69810219 | shiftsize 73 p value 1e-5 -w |
| H2BK12ac | GSM667610, GSM667611 | 58516386 | shiftsize 73 p value 1e-5 -w |
| H2BK120ac | GSM752962 | 10003890 | shiftsize 73 p value 1e-5 -w |
| H2AK5ac | GSM667608, GSM667609 | 56614669 | shiftsize 73 p value 1e-5 -w |
| H2A.Z | GSM807391 | 51808653 | shiftsize 73 p value 1e-5 -w |

**Table S5: ChIP-seq datasets for the histone modifications used in this study** (98+10 datasets from GSE17312, GSE16368 or GSE16256)
