# Supplementary material for: An annotated list of bivalent chromatin regions in human ES cells: a new tool for cancer epigenetic research
Source: Oncotarget. 2016 Dec 1;8(3):4110–24. doi: 10.18632/oncotarget.13746 (PMC5354816; doi:10.18632/oncotarget.13746)
Supplement: Supplementary file 4 [file oncotarget-08-4110-s004.docx]

| H1 | | | | | |
| --- | --- | --- | --- | --- | --- |
| Experiment | Datasets | Macs parameters | Experiment | Datasets | Macs parameters |
| ATF2 | GSM1010742 | shiftsize 73 p value 1e-5 -w | NRSF | GSM803365 | shiftsize 73 p value 1e-5 -w |
| ATF3 | GSM803512 | shiftsize 73 p value 1e-5 -w | P300 | GSM803542,GSM1003513 | shiftsize 73 p value 1e-5 -w |
| BACH1 | GSM935580 | shiftsize 73 p value 1e-5 -w | PHF8 | GSM1003509,GSM831037 | shiftsize 73 p value 1e-5 -w |
| BCL11A | GSM803396,GSM803476 | shiftsize 73 p value 1e-5 -w | POLII | GSM803366,GSM822300 | shiftsize 73 p value 1e-5 -w |
| BRCA1 | GSM935517 | shiftsize 73 p value 1e-5 -w | POLII_S5P | GSM803484 | shiftsize 73 p value 1e-5 -w |
| c-JUN | GSM935614 | shiftsize 73 p value 1e-5 -w | POU5F1 | GSM803438 | shiftsize 73 p value 1e-5 -w |
| c-MYC | GSM822274,GSM935509 | shiftsize 73 p value 1e-5 -w | RAD21 | GSM803466,GSM935379 | shiftsize 73 p value 1e-5 -w |
| CEBPB | GSM935295 | shiftsize 73 p value 1e-5 -w | RBBP5 | GSM1003584,GSM831038 | shiftsize 73 p value 1e-5 -w |
| CHD1 | GSM1003444,GSM935296 | shiftsize 73 p value 1e-5 -w | RFX5 | GSM935382 | shiftsize 73 p value 1e-5 -w |
| CHD2 | GSM935297 | shiftsize 73 p value 1e-5 -w | RXRA | GSM803506 | shiftsize 73 p value 1e-5 -w |
| CHD7 | GSM1003473,GSM831027 | shiftsize 73 p value 1e-5 -w | SAP30 | GSM1003572,GSM831040 | shiftsize 73 p value 1e-5 -w |
| CREB1 | GSM1010896 | shiftsize 73 p value 1e-5 -w | SIN3A | GSM935289,GSM803428 | shiftsize 73 p value 1e-5 -w |
| CTBP2 | GSM935463 | shiftsize 73 p value 1e-5 -w | SIRT6 | GSM1003451,GSM831041 | shiftsize 73 p value 1e-5 -w |
| CTCF | GSM733672,GSM822297,GSM803419 | shiftsize 73 p value 1e-5 -w | SIX5 | GSM803405 | shiftsize 73 p value 1e-5 -w |
| E2F6 | GSM1010899 | shiftsize 73 p value 1e-5 -w | SP1 | GSM803377 | shiftsize 73 p value 1e-5 -w |
| EGR1 | GSM803430 | shiftsize 73 p value 1e-5 -w | SP2 | GSM1010776 | shiftsize 73 p value 1e-5 -w |
| EZH2 | GSM1003524,GSM831028 | shiftsize 73 p value 1e-5 -w | SP4 | GSM1010743 | shiftsize 73 p value 1e-5 -w |
| FOSL1 | GSM803382 | shiftsize 73 p value 1e-5 -w | SRF | GSM803425 | shiftsize 73 p value 1e-5 -w |
| GABPA | GSM803424 | shiftsize 73 p value 1e-5 -w | SUZ12 | GSM1003573,GSM935352 | shiftsize 73 p value 1e-5 -w |
| GTF2F1 | GSM935581 | shiftsize 73 p value 1e-5 -w | TAF1 | GSM803450 | shiftsize 73 p value 1e-5 -w |
| HDAC2 | GSM1003472,GSM803345 | shiftsize 73 p value 1e-5 -w | TAF7 | GSM803501 | shiftsize 73 p value 1e-5 -w |
| HDAC6 | GSM1003571,GSM831030 | shiftsize 73 p value 1e-5 -w | TBP | GSM935303 | shiftsize 73 p value 1e-5 -w |
| JARID1A | GSM1003446,GSM831031 | shiftsize 73 p value 1e-5 -w | TCF12 | GSM803427 | shiftsize 73 p value 1e-5 -w |
| JMJD2A | GSM1003479,GSM831035 | shiftsize 73 p value 1e-5 -w | TEAD4 | GSM1010845 | shiftsize 73 p value 1e-5 -w |
| JUND | GSM803529,GSM935434 | shiftsize 73 p value 1e-5 -w | USF1 | GSM803426 | shiftsize 73 p value 1e-5 -w |
| MAFK | GSM935292 | shiftsize 73 p value 1e-5 -w | USF2 | GSM935380 | shiftsize 73 p value 1e-5 -w |
| MAX | GSM1010898,GSM935348 | shiftsize 73 p value 1e-5 -w | YY1 | GSM803513,GSM956127 | shiftsize 73 p value 1e-5 -w |
| MXI1 | GSM935293 | shiftsize 73 p value 1e-5 -w | ZNF143 | GSM935514 | shiftsize 73 p value 1e-5 -w |
| NANOG | GSM803437 | shiftsize 73 p value 1e-5 -w | ZNF274 | GSM1003619 | shiftsize 73 p value 1e-5 -w |
| NRF1 | GSM935308 | shiftsize 73 p value 1e-5 -w |  |  |  |

**Table S6: ChIP-seq datasets for transcription factors in the hESC line H1 used in this study**.
