# Supplementary material for: An annotated list of bivalent chromatin regions in human ES cells: a new tool for cancer epigenetic research
Source: Oncotarget. 2016 Dec 1;8(3):4110–24. doi: 10.18632/oncotarget.13746 (PMC5354816; doi:10.18632/oncotarget.13746)
Supplement: Supplementary file 5 [file oncotarget-08-4110-s005.docx]

|  |  |  | Infinium MethylationEPIC probes | | | | | Covered by at least one probe | Mean coverage  (probes) |
| --- | --- | --- | --- | --- | --- | --- | --- | --- | --- |
|  |  |  | CpG islands | Shore | Shelf | Other contexts | All Probes |  |  |
| HC bivalent | **CpG Island** | Promoter | 35681 | 22754 | 624 | 81 | 59140 | 4074/99.9% | 14.5 |
|  |  | Gene body | 2309 | 1495 | 13 | 0 | 3817 | 489/99.6% | 7.8 |
|  |  | Intergenic | 2977 | 2069 | 55 | 7 | 5108 | 653/99.2% | 7.8 |
|  |  | **Total** | **40967** | **26318** | **692** | **88** | **68065** | **5216/99.8%** | **13.0** |
|  | **Non- CpG island** | Promoter | 0 | 211 | 188 | 1761 | 2160 | 247/98.8% | 8.7 |
|  |  | Gene body | 0 | 30 | 59 | 275 | 364 | 91/94.8% | 4 |
|  |  | Intergenic | 0 | 22 | 99 | 601 | 722 | 184/96.3% | 3.9 |
|  |  | **Total** | **0** | **263** | **346** | **2637** | **3246** | **522/97.2%** | **6.2** |
|  | **Total** | | **40967** | **26581** | **1038** | **2725** | **71311** | **5738/99.5%** | **12.4** |
| HC H3K4me3 | **CpG Island** | Promoter | 57679 | 39591 | 1477 | 172 | 98919 | 7284/99.8% | 13.6 |
|  |  | Gene body | 789 | 503 | 9 | 0 | 1301 | 244/99.6% | 5.3 |
|  |  | Intergenic | 891 | 563 | 15 | 12 | 1481 | 259/98.9% | 5.7 |
|  |  | **Total** | **59359** | **40657** | **1501** | **184** | **101701** | 7787/99.8% | 13.1 |
|  | **Non- CpG island** | Promoter | 0 | 1362 | 710 | 7494 | 9566 | 1449/91.8% | 6.6 |
|  |  | Gene body | 0 | 231 | 290 | 1572 | 2093 | 1083/76.4% | 1.9 |
|  |  | Intergenic | 0 | 50 | 86 | 1511 | 1647 | 843/72.1% | 2 |
|  |  | **Total** | **0** | **1643** | **1086** | **10577** | **13306** | **3375/81.1%** | **3.9** |
|  | **Total** | | **59359** | **42300** | **2587** | **10761** | **115007** | **11162/93.3%** | **10.3** |
| HC H3K27me3 | **CpG Island** | Promoter | 771 | 1090 | 280 | 124 | 2265 | 247/99.6% | 9.2 |
|  |  | Gene body | 662 | 717 | 185 | 144 | 1708 | 226/99.1% | 7.6 |
|  |  | Intergenic | 441 | 508 | 107 | 16 | 1072 | 159/97% | 6.7 |
|  |  | **Total** | **1874** | **2315** | **572** | **284** | **5045** | **632/**98.8% | **8** |
|  | **Non- CpG island** | Promoter | 0 | 4976 | 3351 | 5730 | 14057 | 3361/89% | 4.2 |
|  |  | Gene body | 0 | 2354 | 2760 | 4352 | 9466 | 4020/71.1% | 2.4 |
|  |  | Intergenic | 1 | 1914 | 2264 | 4268 | 8447 | 4012/63.8% | 2.1 |
|  |  | **Total** | **1** | **9244** | **8375** | **14350** | **31970** | **11393/72.5%** | **2.8** |
|  | **Total** | | **1875** | **11559** | **8947** | **14634** | **37015** | **12025/73.5%** | **3.1** |
|  |  |  | Infinium HumanMethylation450 probes | | | | | Covered by at least one probe | Mean coverage  (probes) |
|  |  |  | CpG islands | Shore | Shelf | Other contexts | All Probes |  |  |
| HC bivalent | **CpG Island** | Promoter | 32676 | 17720 | 504 | 67 | 50967 | 4072/99.8% | 12.5 |
|  |  | Gene body | 2417 | 1371 | 13 | 0 | 3801 | 488/99.4% | 7.8 |
|  |  | Intergenic | 3076 | 1913 | 53 | 5 | 5047 | 651/98.9% | 7.8 |
|  |  | **Total** | **38169** | **21004** | **570** | **72** | **59815** | **5211/99.7%** | **11.5** |
|  | **Non- CpG island** | Promoter | 0 | 153 | 134 | 1280 | 1567 | 240/96% | 6.5 |
|  |  | Gene body | 0 | 30 | 57 | 235 | 322 | 80/83.3% | 4 |
|  |  | Intergenic | 0 | 21 | 95 | 537 | 653 | 171/89.5% | 3.8 |
|  |  | **Total** | **0** | **204** | **286** | **2052** | **2542** | **491/91.4%** | **5.2** |
|  | **Total** | | **38169** | **21208** | **856** | **2124** | **62357** | **5702/98.9%** | **10.9** |
| HC H3K4me3 | **CpG Island** | Promoter | 51301 | 27452 | 1087 | 89 | 79929 | **7282/99.8%** | **11** |
|  |  | Gene body | 808 | 436 | 8 | 0 | 1252 | 243/99.2% | 5.2 |
|  |  | Intergenic | 919 | 480 | 13 | 14 | 1426 | 259/98.9% | 5.5 |
|  |  | **Total** | **53028** | **28368** | **1108** | **103** | **82607** | **7784/99.8%** | **10.6** |
|  | **Non- CpG island** | Promoter | 0 | 878 | 494 | 5073 | 6445 | 1288/81.6% | 5 |
|  |  | Gene body | 0 | 180 | 234 | 919 | 1333 | 839/59.2% | 1.6 |
|  |  | Intergenic | 0 | 40 | 62 | 1011 | 1113 | 627/53.6% | 1.8 |
|  |  | **Total** | **0** | **1098** | **790** | **7003** | **8891** | **2754/66.1%** | **3.2** |
|  | **Total** | | **53028** | **29466** | **1898** | **7106** | **91498** | **10538/88.1%** | **8.7** |
| HC H3K27me3 | **CpG Island** | Promoter | 767 | 801 | 210 | 61 | 1839 | 247/99.6% | 7.4 |
|  |  | Gene body | 685 | 626 | 166 | 112 | 1589 | 226/99.1% | 7 |
|  |  | Intergenic | 464 | 464 | 89 | 13 | 1030 | 160/97.6% | 6.4 |
|  |  | **Total** | **1916** | **1891** | **465** | **186** | **4458** | **633/98.6%** | **7** |
|  | **Non- CpG island** | Promoter | 0 | 2657 | 2261 | 2279 | 7197 | 2701/71.5% | 2.7 |
|  |  | Gene body | 0 | 1737 | 2200 | 1739 | 5676 | 3014/53.3% | 1.8 |
|  |  | Intergenic | 1 | 1293 | 1832 | 1575 | 4701 | 2762/43.9% | 1.7 |
|  |  | **Total** | **1** | **5687** | **6293** | **5593** | **17574** | **8477/53.9%** | **2.1** |
|  | **Total** | | **1917** | **7578** | **6758** | **5779** | **22032** | **9110/55.7%** | **2.4** |

**Table S7: Details of the coverage for the EPIC (upper table) and HM450K (lower table) arrays.**

HC, high confidence.
