# Supplementary material for: An annotated list of bivalent chromatin regions in human ES cells: a new tool for cancer epigenetic research
Source: Oncotarget. 2016 Dec 1;8(3):4110–24. doi: 10.18632/oncotarget.13746 (PMC5354816; doi:10.18632/oncotarget.13746)
Supplement: Supplementary file 6 [file oncotarget-08-4110-s006.docx]

| BRCA n=90 couples | TN | TCGA-A7-A0D9-01 | TCGA-A7-A13E-01 | TCGA-A7-A13F-01 | TCGA-A7-A13G-01 | TCGA-AC-A23H-01 | TCGA-AC-A2FB-01 |
| --- | --- | --- | --- | --- | --- | --- | --- |
|  |  | TCGA-AC-A2FF-01 | TCGA-AC-A2FG-01 | TCGA-AC-A2FM-01 | TCGA-BH-A0AU-01 | TCGA-BH-A0AZ-01 | TCGA-BH-A0B3-01 |
|  |  | TCGA-BH-A0B8-01 | TCGA-BH-A0BA-01 | TCGA-BH-A0BC-01 | TCGA-BH-A0BF-01 | TCGA-BH-A0BJ-01 | TCGA-BH-A0BM-01 |
|  |  | TCGA-BH-A0BS-01 | TCGA-BH-A0BT-01 | TCGA-BH-A0BZ-01 | TCGA-BH-A0C0-01 | TCGA-BH-A0C3-01 | TCGA-BH-A0DG-01 |
|  |  | TCGA-BH-A0DH-01 | TCGA-BH-A0DI-01 | TCGA-BH-A0DK-01 | TCGA-BH-A0DP-01 | TCGA-BH-A0DQ-01 | TCGA-BH-A0DV-01 |
|  |  | TCGA-BH-A0E0-01 | TCGA-BH-A0E1-01 | TCGA-BH-A0H7-01 | TCGA-BH-A0H9-01 | TCGA-BH-A0HA-01 | TCGA-BH-A0HK-01 |
|  |  | TCGA-BH-A1EN-01 | TCGA-BH-A1EO-01 | TCGA-BH-A1ES-01 | TCGA-BH-A1ET-01 | TCGA-BH-A1EU-01 | TCGA-BH-A1EV-01 |
|  |  | TCGA-BH-A1EW-01 | TCGA-BH-A1EY-01 | TCGA-BH-A1F0-01 | TCGA-BH-A1F2-01 | TCGA-BH-A1F5-01 | TCGA-BH-A1F6-01 |
|  |  | TCGA-BH-A1F8-01 | TCGA-BH-A1FB-01 | TCGA-BH-A1FC-01 | TCGA-BH-A1FD-01 | TCGA-BH-A1FE-01 | TCGA-BH-A1FG-01 |
|  |  | TCGA-BH-A1FH-01 | TCGA-BH-A1FJ-01 | TCGA-BH-A1FM-01 | TCGA-BH-A1FN-01 | TCGA-BH-A1FR-01 | TCGA-BH-A203-01 |
|  |  | TCGA-BH-A204-01 | TCGA-BH-A208-01 | TCGA-BH-A209-01 | TCGA-E2-A15I-01 | TCGA-E2-A15K-01 | TCGA-E2-A1B5-01 |
|  |  | TCGA-E2-A1BC-01 | TCGA-E2-A1IF-01 | TCGA-E2-A1II-01 | TCGA-E2-A1IO-01 | TCGA-E2-A1L7-01 | TCGA-E2-A1LB-01 |
|  |  | TCGA-E2-A1LI-01 | TCGA-E2-A1LS-01 | TCGA-E9-A1N4-01 | TCGA-E9-A1N5-01 | TCGA-E9-A1N6-01 | TCGA-E9-A1N8-01 |
|  |  | TCGA-E9-A1NA-01 | TCGA-E9-A1ND-01 | TCGA-E9-A1NE-01 | TCGA-E9-A1NF-01 | TCGA-E9-A1NG-01 | TCGA-E9-A1R7-01 |
|  |  | TCGA-E9-A1RB-01 | TCGA-E9-A1RC-01 | TCGA-E9-A1RD-01 | TCGA-E9-A1RF-01 | TCGA-E9-A1RH-01 | TCGA-E9-A1RI-01 |
|  | NT | TCGA-A7-A0D9-11 | TCGA-A7-A13E-11 | TCGA-A7-A13F-11 | TCGA-A7-A13G-11 | TCGA-AC-A23H-11 | TCGA-AC-A2FB-11 |
|  |  | TCGA-AC-A2FF-11 | TCGA-AC-A2FG-11 | TCGA-AC-A2FM-11 | TCGA-BH-A0AU-11 | TCGA-BH-A0AZ-11 | TCGA-BH-A0B3-11 |
|  |  | TCGA-BH-A0B8-11 | TCGA-BH-A0BA-11 | TCGA-BH-A0BC-11 | TCGA-BH-A0BF-11 | TCGA-BH-A0BJ-11 | TCGA-BH-A0BM-11 |
|  |  | TCGA-BH-A0BS-11 | TCGA-BH-A0BT-11 | TCGA-BH-A0BZ-11 | TCGA-BH-A0C0-11 | TCGA-BH-A0C3-11 | TCGA-BH-A0DG-11 |
|  |  | TCGA-BH-A0DH-11 | TCGA-BH-A0DI-11 | TCGA-BH-A0DK-11 | TCGA-BH-A0DP-11 | TCGA-BH-A0DQ-11 | TCGA-BH-A0DV-11 |
|  |  | TCGA-BH-A0E0-11 | TCGA-BH-A0E1-11 | TCGA-BH-A0H7-11 | TCGA-BH-A0H9-11 | TCGA-BH-A0HA-11 | TCGA-BH-A0HK-11 |
|  |  | TCGA-BH-A1EN-11 | TCGA-BH-A1EO-11 | TCGA-BH-A1ES-11 | TCGA-BH-A1ET-11 | TCGA-BH-A1EU-11 | TCGA-BH-A1EV-11 |
|  |  | TCGA-BH-A1EW-11 | TCGA-BH-A1EY-11 | TCGA-BH-A1F0-11 | TCGA-BH-A1F2-11 | TCGA-BH-A1F5-11 | TCGA-BH-A1F6-11 |
|  |  | TCGA-BH-A1F8-11 | TCGA-BH-A1FB-11 | TCGA-BH-A1FC-11 | TCGA-BH-A1FD-11 | TCGA-BH-A1FE-11 | TCGA-BH-A1FG-11 |
|  |  | TCGA-BH-A1FH-11 | TCGA-BH-A1FJ-11 | TCGA-BH-A1FM-11 | TCGA-BH-A1FN-11 | TCGA-BH-A1FR-11 | TCGA-BH-A203-11 |
|  |  | TCGA-BH-A204-11 | TCGA-BH-A208-11 | TCGA-BH-A209-11 | TCGA-E2-A15I-11 | TCGA-E2-A15K-11 | TCGA-E2-A1B5-11 |
|  |  | TCGA-E2-A1BC-11 | TCGA-E2-A1IF-11 | TCGA-E2-A1II-11 | TCGA-E2-A1IO-11 | TCGA-E2-A1L7-11 | TCGA-E2-A1LB-11 |
|  |  | TCGA-E2-A1LI-11 | TCGA-E2-A1LS-11 | TCGA-E9-A1N4-11 | TCGA-E9-A1N5-11 | TCGA-E9-A1N6-11 | TCGA-E9-A1N8-11 |
|  |  | TCGA-E9-A1NA-11 | TCGA-E9-A1ND-11 | TCGA-E9-A1NE-11 | TCGA-E9-A1NF-11 | TCGA-E9-A1NG-11 | TCGA-E9-A1R7-11 |
|  |  | TCGA-E9-A1RB-11 | TCGA-E9-A1RC-11 | TCGA-E9-A1RD-11 | TCGA-E9-A1RF-11 | TCGA-E9-A1RH-11 | TCGA-E9-A1RI-11 |
|  |  |  |  |  |  |  |  |
| BLCA n=21 couples | TN | TCGA-BL-A13J-01 | TCGA-BT-A20J-01 | TCGA-BT-A20N-01 | TCGA-BT-A20P-01 | TCGA-BT-A20R-01 | TCGA-BT-A20U-01 |
|  |  | TCGA-BT-A20V-01 | TCGA-BT-A20W-01 | TCGA-BT-A20X-01 | TCGA-BT-A2LA-01 | TCGA-CU-A0YN-01 | TCGA-CU-A0YR-01 |
|  |  | TCGA-GC-A3BM-01 | TCGA-GC-A3WC-01 | TCGA-GC-A6I3-01 | TCGA-GD-A2C5-01 | TCGA-GD-A3OP-01 | TCGA-GD-A3OQ-01 |
|  |  | TCGA-K4-A3WV-01 | TCGA-K4-A54R-01 | TCGA-K4-A5RI-01 |  |  |  |
|  | NT | TCGA-BL-A13J-11 | TCGA-BT-A20J-11 | TCGA-BT-A20N-11 | TCGA-BT-A20P-11 | TCGA-BT-A20R-11 | TCGA-BT-A20U-11 |
|  |  | TCGA-BT-A20V-11 | TCGA-BT-A20W-11 | TCGA-BT-A20X-11 | TCGA-BT-A2LA-11 | TCGA-CU-A0YN-11 | TCGA-CU-A0YR-11 |
|  |  | TCGA-GC-A3BM-11 | TCGA-GC-A3WC-11 | TCGA-GC-A6I3-11 | TCGA-GD-A2C5-11 | TCGA-GD-A3OP-11 | TCGA-GD-A3OQ-11 |
|  |  | TCGA-K4-A3WV-11 | TCGA-K4-A54R-11 | TCGA-K4-A5RI-11 |  |  |  |
|  |  |  |  |  |  |  |  |
| COAD n=38 couples | TN | TCGA-A6-2671-01 | TCGA-A6-2675-01 | TCGA-A6-2679-01 | TCGA-A6-2680-01 | TCGA-A6-2681-01 | TCGA-A6-2682-01 |
|  |  | TCGA-A6-2684-01 | TCGA-A6-2685-01 | TCGA-A6-2686-01 | TCGA-A6-4107-01 | TCGA-A6-5667-01 | TCGA-AA-3488-01 |
|  |  | TCGA-AA-3492-01 | TCGA-AA-3494-01 | TCGA-AA-3495-01 | TCGA-AA-3502-01 | TCGA-AA-3506-01 | TCGA-AA-3509-01 |
|  |  | TCGA-AA-3510-01 | TCGA-AA-3655-01 | TCGA-AA-3660-01 | TCGA-AA-3663-01 | TCGA-AA-3697-01 | TCGA-AA-3712-01 |
|  |  | TCGA-AA-3713-01 | TCGA-AZ-6598-01 | TCGA-AZ-6599-01 | TCGA-AZ-6600-01 | TCGA-AZ-6601-01 | TCGA-G4-6295-01 |
|  |  | TCGA-G4-6297-01 | TCGA-G4-6298-01 | TCGA-G4-6302-01 | TCGA-G4-6311-01 | TCGA-G4-6314-01 | TCGA-G4-6320-01 |
|  |  | TCGA-G4-6322-01 | TCGA-G4-6625-01 |  |  |  |  |
|  | NT | TCGA-A6-2671-11 | TCGA-A6-2675-11 | TCGA-A6-2679-11 | TCGA-A6-2680-11 | TCGA-A6-2681-11 | TCGA-A6-2682-11 |
|  |  | TCGA-A6-2684-11 | TCGA-A6-2685-11 | TCGA-A6-2686-11 | TCGA-A6-4107-11 | TCGA-A6-5667-11 | TCGA-AA-3488-11 |
|  |  | TCGA-AA-3492-11 | TCGA-AA-3494-11 | TCGA-AA-3495-11 | TCGA-AA-3502-11 | TCGA-AA-3506-11 | TCGA-AA-3509-11 |
|  |  | TCGA-AA-3510-11 | TCGA-AA-3655-11 | TCGA-AA-3660-11 | TCGA-AA-3663-11 | TCGA-AA-3697-11 | TCGA-AA-3712-11 |
|  |  | TCGA-AA-3713-11 | TCGA-AZ-6598-11 | TCGA-AZ-6599-11 | TCGA-AZ-6600-11 | TCGA-AZ-6601-11 | TCGA-G4-6295-11 |
|  |  | TCGA-G4-6297-11 | TCGA-G4-6298-11 | TCGA-G4-6302-11 | TCGA-G4-6311-11 | TCGA-G4-6314-11 | TCGA-G4-6320-11 |
|  |  | TCGA-G4-6322-11 | TCGA-G4-6625-11 |  |  |  |  |
|  |  |  |  |  |  |  |  |
| HNSC n=50 couples | TN | TCGA-CV-5430-01 | TCGA-CV-5431-01 | TCGA-CV-5432-01 | TCGA-CV-5434-01 | TCGA-CV-5435-01 | TCGA-CV-5436-01 |
|  |  | TCGA-CV-5439-01 | TCGA-CV-5440-01 | TCGA-CV-5441-01 | TCGA-CV-5442-01 | TCGA-CV-5443-01 | TCGA-CV-5444-01 |
|  |  | TCGA-CV-5966-01 | TCGA-CV-5970-01 | TCGA-CV-5971-01 | TCGA-CV-5973-01 | TCGA-CV-5976-01 | TCGA-CV-5977-01 |
|  |  | TCGA-CV-5978-01 | TCGA-CV-5979-01 | TCGA-CV-6003-01 | TCGA-CV-6433-01 | TCGA-CV-6436-01 | TCGA-CV-6441-01 |
|  |  | TCGA-CV-6933-01 | TCGA-CV-6934-01 | TCGA-CV-6935-01 | TCGA-CV-6936-01 | TCGA-CV-6938-01 | TCGA-CV-6939-01 |
|  |  | TCGA-CV-6943-01 | TCGA-CV-6951-01 | TCGA-CV-6952-01 | TCGA-CV-6953-01 | TCGA-CV-6954-01 | TCGA-CV-6955-01 |
|  |  | TCGA-CV-6956-01 | TCGA-CV-6959-01 | TCGA-CV-6961-01 | TCGA-CV-6962-01 | TCGA-CV-7089-01 | TCGA-CV-7101-01 |
|  |  | TCGA-CV-7103-01 | TCGA-CV-7178-01 | TCGA-CV-7235-01 | TCGA-CV-7238-01 | TCGA-CV-7245-01 | TCGA-CV-7250-01 |
|  |  | TCGA-CV-7255-01 | TCGA-CV-7263-01 |  |  |  |  |
|  | NT | TCGA-CV-5430-11 | TCGA-CV-5431-11 | TCGA-CV-5432-11 | TCGA-CV-5434-11 | TCGA-CV-5435-11 | TCGA-CV-5436-11 |
|  |  | TCGA-CV-5439-11 | TCGA-CV-5440-11 | TCGA-CV-5441-11 | TCGA-CV-5442-11 | TCGA-CV-5443-11 | TCGA-CV-5444-11 |
|  |  | TCGA-CV-5966-11 | TCGA-CV-5970-11 | TCGA-CV-5971-11 | TCGA-CV-5973-11 | TCGA-CV-5976-11 | TCGA-CV-5977-11 |
|  |  | TCGA-CV-5978-11 | TCGA-CV-5979-11 | TCGA-CV-6003-11 | TCGA-CV-6433-11 | TCGA-CV-6436-11 | TCGA-CV-6441-11 |
|  |  | TCGA-CV-6933-11 | TCGA-CV-6934-11 | TCGA-CV-6935-11 | TCGA-CV-6936-11 | TCGA-CV-6938-11 | TCGA-CV-6939-11 |
|  |  | TCGA-CV-6943-11 | TCGA-CV-6951-11 | TCGA-CV-6952-11 | TCGA-CV-6953-11 | TCGA-CV-6954-11 | TCGA-CV-6955-11 |
|  |  | TCGA-CV-6956-11 | TCGA-CV-6959-11 | TCGA-CV-6961-11 | TCGA-CV-6962-11 | TCGA-CV-7089-11 | TCGA-CV-7101-11 |
|  |  | TCGA-CV-7103-11 | TCGA-CV-7178-11 | TCGA-CV-7235-11 | TCGA-CV-7238-11 | TCGA-CV-7245-11 | TCGA-CV-7250-11 |
|  |  | TCGA-CV-7255-11 | TCGA-CV-7263-11 |  |  |  |  |
|  |  |  |  |  |  |  |  |
| KIRP n=45 couples | TN | TCGA-A4-7288-01 | TCGA-A4-7585-01 | TCGA-A4-7732-01 | TCGA-B1-A47M-01 | TCGA-BQ-5875-01 | TCGA-BQ-5876-01 |
|  |  | TCGA-BQ-5877-01 | TCGA-BQ-5878-01 | TCGA-BQ-5879-01 | TCGA-BQ-5880-01 | TCGA-BQ-5881-01 | TCGA-BQ-5882-01 |
|  |  | TCGA-BQ-5883-01 | TCGA-BQ-5884-01 | TCGA-BQ-5885-01 | TCGA-BQ-5886-01 | TCGA-BQ-5887-01 | TCGA-BQ-5888-01 |
|  |  | TCGA-BQ-5889-01 | TCGA-BQ-5890-01 | TCGA-BQ-5891-01 | TCGA-BQ-5892-01 | TCGA-BQ-5893-01 | TCGA-BQ-5894-01 |
|  |  | TCGA-BQ-7044-01 | TCGA-BQ-7045-01 | TCGA-BQ-7046-01 | TCGA-BQ-7048-01 | TCGA-BQ-7049-01 | TCGA-BQ-7050-01 |
|  |  | TCGA-BQ-7051-01 | TCGA-BQ-7053-01 | TCGA-BQ-7055-01 | TCGA-BQ-7056-01 | TCGA-BQ-7058-01 | TCGA-BQ-7059-01 |
|  |  | TCGA-BQ-7060-01 | TCGA-BQ-7061-01 | TCGA-BQ-7062-01 | TCGA-DZ-6131-01 | TCGA-DZ-6132-01 | TCGA-DZ-6133-01 |
|  |  | TCGA-DZ-6134-01 | TCGA-DZ-6135-01 | TCGA-GL-6846-01 |  |  |  |
|  | NT | TCGA-A4-7288-11 | TCGA-A4-7585-11 | TCGA-A4-7732-11 | TCGA-B1-A47M-11 | TCGA-BQ-5875-11 | TCGA-BQ-5876-11 |
|  |  | TCGA-BQ-5877-11 | TCGA-BQ-5878-11 | TCGA-BQ-5879-11 | TCGA-BQ-5880-11 | TCGA-BQ-5881-11 | TCGA-BQ-5882-11 |
|  |  | TCGA-BQ-5883-11 | TCGA-BQ-5884-11 | TCGA-BQ-5885-11 | TCGA-BQ-5886-11 | TCGA-BQ-5887-11 | TCGA-BQ-5888-11 |
|  |  | TCGA-BQ-5889-11 | TCGA-BQ-5890-11 | TCGA-BQ-5891-11 | TCGA-BQ-5892-11 | TCGA-BQ-5893-11 | TCGA-BQ-5894-11 |
|  |  | TCGA-BQ-7044-11 | TCGA-BQ-7045-11 | TCGA-BQ-7046-11 | TCGA-BQ-7048-11 | TCGA-BQ-7049-11 | TCGA-BQ-7050-11 |
|  |  | TCGA-BQ-7051-11 | TCGA-BQ-7053-11 | TCGA-BQ-7055-11 | TCGA-BQ-7056-11 | TCGA-BQ-7058-11 | TCGA-BQ-7059-11 |
|  |  | TCGA-BQ-7060-11 | TCGA-BQ-7061-11 | TCGA-BQ-7062-11 | TCGA-DZ-6131-11 | TCGA-DZ-6132-11 | TCGA-DZ-6133-11 |
|  |  | TCGA-DZ-6134-11 | TCGA-DZ-6135-11 | TCGA-GL-6846-11 |  |  |  |
|  |  |  |  |  |  |  |  |
| LIHC n=50 couples | TN | TCGA-BC-A10Q-01 | TCGA-BC-A10R-01 | TCGA-BC-A10S-01 | TCGA-BC-A10T-01 | TCGA-BC-A10U-01 | TCGA-BC-A10W-01 |
|  |  | TCGA-BC-A10X-01 | TCGA-BC-A10Y-01 | TCGA-BC-A10Z-01 | TCGA-BC-A110-01 | TCGA-BC-A112-01 | TCGA-BC-A216-01 |
|  |  | TCGA-BD-A2L6-01 | TCGA-BD-A3EP-01 | TCGA-DD-A113-01 | TCGA-DD-A114-01 | TCGA-DD-A115-01 | TCGA-DD-A116-01 |
|  |  | TCGA-DD-A118-01 | TCGA-DD-A119-01 | TCGA-DD-A11A-01 | TCGA-DD-A11B-01 | TCGA-DD-A11C-01 | TCGA-DD-A11D-01 |
|  |  | TCGA-DD-A1E9-01 | TCGA-DD-A1EB-01 | TCGA-DD-A1EC-01 | TCGA-DD-A1ED-01 | TCGA-DD-A1EE-01 | TCGA-DD-A1EF-01 |
|  |  | TCGA-DD-A1EG-01 | TCGA-DD-A1EH-01 | TCGA-DD-A1EI-01 | TCGA-DD-A1EJ-01 | TCGA-DD-A1EL-01 | TCGA-DD-A39V-01 |
|  |  | TCGA-DD-A39W-01 | TCGA-DD-A39X-01 | TCGA-DD-A39Z-01 | TCGA-DD-A3A1-01 | TCGA-DD-A3A2-01 | TCGA-DD-A3A3-01 |
|  |  | TCGA-EP-A12J-01 | TCGA-EP-A26S-01 | TCGA-ES-A2HS-01 | TCGA-ES-A2HT-01 | TCGA-FV-A23B-01 | TCGA-FV-A2QR-01 |
|  |  | TCGA-G3-A25W-01 | TCGA-G3-A25X-01 |  |  |  |  |
|  | NT | TCGA-BC-A10Q-11 | TCGA-BC-A10R-11 | TCGA-BC-A10S-11 | TCGA-BC-A10T-11 | TCGA-BC-A10U-11 | TCGA-BC-A10W-11 |
|  |  | TCGA-BC-A10X-11 | TCGA-BC-A10Y-11 | TCGA-BC-A10Z-11 | TCGA-BC-A110-11 | TCGA-BC-A112-11 | TCGA-BC-A216-11 |
|  |  | TCGA-BD-A2L6-11 | TCGA-BD-A3EP-11 | TCGA-DD-A113-11 | TCGA-DD-A114-11 | TCGA-DD-A115-11 | TCGA-DD-A116-11 |
|  |  | TCGA-DD-A118-11 | TCGA-DD-A119-11 | TCGA-DD-A11A-11 | TCGA-DD-A11B-11 | TCGA-DD-A11C-11 | TCGA-DD-A11D-11 |
|  |  | TCGA-DD-A1E9-11 | TCGA-DD-A1EB-11 | TCGA-DD-A1EC-11 | TCGA-DD-A1ED-11 | TCGA-DD-A1EE-11 | TCGA-DD-A1EF-11 |
|  |  | TCGA-DD-A1EG-11 | TCGA-DD-A1EH-11 | TCGA-DD-A1EI-11 | TCGA-DD-A1EJ-11 | TCGA-DD-A1EL-11 | TCGA-DD-A39V-11 |
|  |  | TCGA-DD-A39W-11 | TCGA-DD-A39X-11 | TCGA-DD-A39Z-11 | TCGA-DD-A3A1-11 | TCGA-DD-A3A2-11 | TCGA-DD-A3A3-11 |
|  |  | TCGA-EP-A12J-11 | TCGA-EP-A26S-11 | TCGA-ES-A2HS-11 | TCGA-ES-A2HT-11 | TCGA-FV-A23B-11 | TCGA-FV-A2QR-11 |
|  |  | TCGA-G3-A25W-11 | TCGA-G3-A25X-11 |  |  |  |  |
|  |  |  |  |  |  |  |  |
| LUAD n=29 couples | TN | TCGA-73-4676-11 | TCGA-73-4658-11 | TCGA-50-6594-11 | TCGA-50-6593-11 | TCGA-50-6592-11 | TCGA-50-6591-11 |
|  |  | TCGA-50-5939-11 | TCGA-50-5936-11 | TCGA-50-5935-11 | TCGA-50-5933-11 | TCGA-50-5932-11 | TCGA-50-5931-11 |
|  |  | TCGA-50-5930-11 | TCGA-49-6745-11 | TCGA-49-4488-11 | TCGA-44-6778-11 | TCGA-44-6148-11 | TCGA-44-6147-11 |
|  |  | TCGA-44-6146-11 | TCGA-44-6145-11 | TCGA-44-6144-11 | TCGA-44-5645-11 | TCGA-44-5643-11 | TCGA-44-2668-11 |
|  |  | TCGA-44-2665-11 | TCGA-44-2656-11 | TCGA-38-4632-11 | TCGA-38-4631-11 | TCGA-05-5420-11 |  |
|  | NT | TCGA-73-4676-01 | TCGA-73-4658-01 | TCGA-50-6594-01 | TCGA-50-6593-01 | TCGA-50-6592-01 | TCGA-50-6591-01 |
|  |  | TCGA-50-5939-01 | TCGA-50-5936-01 | TCGA-50-5935-01 | TCGA-50-5933-01 | TCGA-50-5932-01 | TCGA-50-5931-01 |
|  |  | TCGA-50-5930-01 | TCGA-49-6745-01 | TCGA-49-4488-01 | TCGA-44-6778-01 | TCGA-44-6148-01 | TCGA-44-6147-01 |
|  |  | TCGA-44-6146-01 | TCGA-44-6145-01 | TCGA-44-6144-01 | TCGA-44-5645-01 | TCGA-44-5643-01 | TCGA-44-2668-01 |
|  |  | TCGA-44-2665-01 | TCGA-44-2656-01 | TCGA-38-4632-01 | TCGA-38-4631-01 | TCGA-05-5420-01 |  |
|  |  |  |  |  |  |  |  |
| LUSC n=40 couples | TN | TCGA-18-3417-01 | TCGA-18-4721-01 | TCGA-18-5592-01 | TCGA-18-5595-01 | TCGA-22-4599-01 | TCGA-22-4601-01 |
|  |  | TCGA-22-4613-01 | TCGA-22-5471-01 | TCGA-22-5472-01 | TCGA-22-5473-01 | TCGA-22-5474-01 | TCGA-22-5477-01 |
|  |  | TCGA-22-5478-01 | TCGA-22-5480-01 | TCGA-22-5482-01 | TCGA-22-5485-01 | TCGA-22-5489-01 | TCGA-22-5491-01 |
|  |  | TCGA-22-5492-01 | TCGA-33-4566-01 | TCGA-33-4582-01 | TCGA-33-4583-01 | TCGA-33-4586-01 | TCGA-33-4589-01 |
|  |  | TCGA-34-5929-01 | TCGA-39-5011-01 | TCGA-39-5016-01 | TCGA-39-5019-01 | TCGA-39-5021-01 | TCGA-39-5028-01 |
|  |  | TCGA-39-5029-01 | TCGA-39-5030-01 | TCGA-39-5031-01 | TCGA-39-5034-01 | TCGA-39-5035-01 | TCGA-39-5036-01 |
|  |  | TCGA-39-5037-01 | TCGA-39-5039-01 | TCGA-43-5668-01 | TCGA-43-6771-01 |  |  |
|  | NT | TCGA-18-3417-11 | TCGA-18-4721-11 | TCGA-18-5592-11 | TCGA-18-5595-11 | TCGA-22-4599-11 | TCGA-22-4601-11 |
|  |  | TCGA-22-4613-11 | TCGA-22-5471-11 | TCGA-22-5472-11 | TCGA-22-5473-11 | TCGA-22-5474-11 | TCGA-22-5477-11 |
|  |  | TCGA-22-5478-11 | TCGA-22-5480-11 | TCGA-22-5482-11 | TCGA-22-5485-11 | TCGA-22-5489-11 | TCGA-22-5491-11 |
|  |  | TCGA-22-5492-11 | TCGA-33-4566-11 | TCGA-33-4582-11 | TCGA-33-4583-11 | TCGA-33-4586-11 | TCGA-33-4589-11 |
|  |  | TCGA-34-5929-11 | TCGA-39-5011-11 | TCGA-39-5016-11 | TCGA-39-5019-11 | TCGA-39-5021-11 | TCGA-39-5028-11 |
|  |  | TCGA-39-5029-11 | TCGA-39-5030-11 | TCGA-39-5031-11 | TCGA-39-5034-11 | TCGA-39-5035-11 | TCGA-39-5036-11 |
|  |  | TCGA-39-5037-11 | TCGA-39-5039-11 | TCGA-43-5668-11 | TCGA-43-6771-11 |  |  |

**Table S8: Datasets extracted from the TCGA data portal for cancer methylation analyses**

BLCA = bladder urothelial carcinoma; BRCA = breast invasive carcinoma; COAD = colon adenocarcinoma; HNSC = head-neck squamous cell carcinoma; KIRP = kidney renal papillary cell carcinoma; LIHC = liver hepatocellular carcinoma; LUSC = lung squamous cell carcinoma; LUAD = lung adenocarcinoma; NT = Normal Matched Tumor; TN = Tumor Matched Normal.
