# Supplementary material for: An annotated list of bivalent chromatin regions in human ES cells: a new tool for cancer epigenetic research
Source: Oncotarget. 2016 Dec 1;8(3):4110–24. doi: 10.18632/oncotarget.13746 (PMC5354816; doi:10.18632/oncotarget.13746)
Supplement: Supplementary file 7 [file oncotarget-08-4110-s007.docx]

|  | **BLCA** | | **BRCA** | | **COAD** | | **HNSC** | | **KIRP** | | **LIHC** | | **LUAD** | | **LUSC** | |  |
| --- | --- | --- | --- | --- | --- | --- | --- | --- | --- | --- | --- | --- | --- | --- | --- | --- | --- |
|  | CIMP - | CIMP + | CIMP - | CIMP + | CIMP - | CIMP + | CIMP - | CIMP + | CIMP - | CIMP + | CIMP - | CIMP + | CIMP - | CIMP + | CIMP - | CIMP + |  |
| Bivalent | 56%  (n=369) | 64%  (n=1273) | 50%  (n=301) | 65%  (n=2692) | 73%  (n=885) | 70%  (n=2935) | 59%  (n=715) | 66%  (n=2017) | 27%  (n=72) | 47%  (n=1210) | 45%  (n=48) | 64%  (n=2419) | 55%  (n=301) | 73%  (n=1964) | 56%  (n=455) | 58%  (n=1453) | Hypermethylated |
| H3K4me3- only | 6%  (n=42) | 4%  (n=117) | 5%  (n=34) | 4%  (n=317) | 8%  (n=99) | 7%  (n=397) | 7%  (n=108) | 6%  (n=292) | 10%  (n=28) | 7%  (n=194) | 9%  (n=10) | 7%  (n=388) | 2%  (n=16) | 2%  (n=114) | 4%  (n=39) | 5%  (n=175) |  |
| H3K27me3- only | 2%  (n=22) | 2%  (n=70) | 2%  (n=15) | 2%  (n=137) | 1%  (n=16) | 1%  (n=95) | 2%  (n=40) | 1%  (n=90) | 3%  (n=9) | 3%  (n=91) | 1%  (n=1) | 1%  (n=70) | 3%  (n=25) | 2%  (n=83) | 3%  (n=37) | 3%  (n=115) |  |
| None | 35% | 30% | 43% | 29% | 18% | 23% | 31% | 26% | 60% | 43% | 46% | 29% | 39% | 23% | 37% | 34% |  |
| Total probes | 1570 | 7248 | 976 | 19052 | 3373 | 23424 | 3246 | 14296 | 445 | 6121 | 160 | 14452 | 1014 | 11318 | 2249 | 10434 |  |
| Bivalent | 3%  (n=32) | 6%  (n=12) | 6%  (n=8) | 6%  (n=17) | 4%  (n=11) | 2%  (n=9) | 2%  (n=6) | 1%  (n=8) | 27%  (n=6) | 14%  (n=14) | 1%  (n=5) | 1%  (n=6) | 1%  (n=2) | 10%  (n=3) | 2%  (n=23) | 7%  (n=76) | Hypomethylated |
| H3K4me3- only | 2%  (n=15) | 2%  (n=4) | 0%  (n=0) | 4%  (n=8) | 3%  (n=8) | 3%  (n=11) | 0%  (n=2) | 1%  (n=7) | 13%  (n=2) | 11%  (n=11) | 1%  (n=4) | 1%  (n=9) | 6%  (n=5) | 10%  (n=2) | 1%  (n=14) | 5%  (n=61) |  |
| H3K27me3- only | 12%  (n=92) | 12%  (n=19) | 8%  (n=8) | 7%  (n=19) | 9%  (n=27) | 5%  (n=20) | 8%  (n=28) | 9%  (n=51) | 3%  (n=1) | 8%  (n=6) | 5%  (n=26) | 9%  (n=49) | 13%  (n=16) | 10%  (n=2) | 11%  (n=93) | 9%  (n=89) |  |
| None | 83% | 79% | 87% | 83% | 83% | 90% | 90% | 89% | 57% | 67% | 93% | 89% | 80% | 69% | 86% | 79% |  |
| Total probes | 1425 | 250 | 200 | 393 | 359 | 534 | 428 | 769 | 30 | 115 | 589 | 892 | 138 | 49 | 1572 | 1756 |  |

**Table S10: Distribution (percentage) of hyper- (upper table) and hypo- (lower table) methylated probes in CIMP-negative (-) and CIMP-positive (+) tumors (eight cancer types), according to the chromatin signature in hESCs**. The number of affected genomic regions is indicated into brackets. BLCA: bladder urothelial carcinoma; BRCA: breast invasive carcinoma; COAD: colon adenocarcinoma; HNSC: head-neck squamous cell carcinoma; KIRP: kidney renal papillary cell carcinoma; LIHC: liver hepatocellular carcinoma; LUSC: lung squamous cell carcinoma; LUAD: lung adenocarcinoma
